# *Buchnera* breaks the specialization of the cotton-specialized aphid (*Aphis gossypii*) by providing nutrition through zucchini

**DOI:** 10.3389/fnut.2023.1128272

**Published:** 2023-03-21

**Authors:** Weili Xu, Weijiao Liu, Jinming Li, Xiangzhen Zhu, Li Wang, Dongyang Li, Kaixin Zhang, Jichao Ji, Xueke Gao, Junyu Luo, Jinjie Cui

**Affiliations:** ^1^State Key Laboratory of Cotton Biology, Institute of Cotton Research, Chinese Academy of Agricultural Sciences, Anyang, Henan, China; ^2^Zhengzhou Research Base, State Key Laboratory of Cotton Biology, School of Agricultural Sciences, Zhengzhou University, Zhengzhou, Henan, China; ^3^Western Agricultural Research Center, Chinese Academy of Agricultural Sciences, Changji, China

**Keywords:** *Aphis gossypii*, *Buchnera*, bacterial communities, host specialization, zucchini

## Abstract

The cotton aphid, *Aphis gossypii* Glover, is a species of polyphagous aphid with many biotypes, and its host transfer has always been the focus of research on the control of cotton aphid. An important factor affecting aphid specialization is the nutritional association with microbial symbionts that provide the host with nutrients lacking in the diet. We analyzed the microbial composition and biodiversity of reared on zucchini for 10 generations (T1–T10) and cotton as a control (CK), by high-throughput Illumina sequencing of 16S ribosomal RNA genes. The findings showed that the change in plant hosts decreased the richness and variety of microbial species. Regardless of whether the plant host is altered or not, *Proteobacteria* and *Firmicutes* are the predominate phyla in cotton-specialized aphid. Additionally, cotton-specialized aphids that live in zucchini had considerably lower relative abundances of non-dominant phyla (*Bacteroidetes*) than cotton hosts. At the genus level the dominant communities were *Buchnera*, *Acinetobacter*, and *Arsenophonus*. The relative abundance of *Buchnera* was significantly higher in aphids reared on zucchini than those on cotton, whereas the opposite was observed for *Acinetobacter*, as well as for some non-dominant communities (*Stenotrophomonas*, *Pseudomons*, *Flavobacterium*, *Novosphingobium*). Collectively, this study clarifies the dynamic changes of symbiotic bacteria in cotton-specialized aphids reared on zucchini for multiple generations. Among them, *Buchnera* is crucial for the cotton-specialized aphid to get nutrients during the transfer of the host and has a favorable impact on the colonization of cotton-specialized aphid populations on zucchini hosts. It not only enriches our understanding of the relationship between the bacterial microbiota of aphids and their adaptability to new hosts, zucchini, but also expands the current body of research on the mechanisms underlying the host shifting ability of cotton-specialized aphids.

## Introduction

*Aphis gossypii* Glover (Hemiptera: Aphididae) is a significant agricultural pest of several crops that causes significant feeding damage and spreads viruses (1). Due to their small size, large base, wide distribution, and complex life history, aphids are able to adapt to most environments, making them an increasingly rampant threat to agricultural and forestry development, and a source of great economic losses (2). As a result, *A. gossypii* has been a major problem in agricultural production for many years. The migration of *A. gossypii* is known to be an important factor affecting cotton aphid control. When *A. gossypii* do not have optimal host plants, they produce winged aphids that migrate to other host plants and use them to supplement their own needs for development (3). Thus, host adaptation is an important condition for the outbreak and spread of *A. gossypii*, and the study of adaptive mechanisms of *A. gossypii* regarding host transfer is of great significance for cotton aphid control.

Host specialization refers to the preference of insects to feed and live on specific host plants and the specificity of long-term feeding under natural conditions. While there are some polyphagous insects that can feed on hundreds of different host plants, less than 10% of plant feeding insects can feed on three or more different host plants (4). Most of these insects tend to feed on several closely related plants, or have a strong preference for a single host plant with a specific chemical composition (5). Studies have demonstrated that cotton aphids can produce population differentiation on different plant hosts, and various specific host biotypes have been identified around the world, including cotton and melon (6, 7). In China, based on host plant preferences and complete mitochondrial sequences, *A. gossypii* has been divided into cotton and cucumber biotypes (8). The two biotypes were unable to form populations after transferring to other plant hosts, and their survival rates drastically dropped, according to host transfer tests (9). However, it has also been shown that specialization is not absolute and that cotton-specialized aphids can migrate to each other through zucchini as bridge hosts. Cotton-specialized aphids can be successfully transferred to cucumber and cotton after several generations of culture on zucchini successfully transferred to cucumber and cotton, with no significant differences from aphids on the original host after transfer (10). However, it is unknown why zucchini can act as an intermediate bridge for host transformation and how cotton-specialized aphids absorb sufficient nutrients during host adaptation.

A very close relationship exists between insects and their endosymbionts, and interactions between *A. gossypii* and their bacterial communities have received increased attention in recent years. aphid endosymbiotic bacteria include primary endosymbionts, such as *Buchnera*, and secondary symbionts which include *Rickettsia spp, Wolbachia spp*, *Hamiltonella, Arsenophonus*, *Acinetobacter*, *Micrococcus*, *Regiella insecticola, Serratia*, and *Pseudomonadaceae*. Different symbiotic bacteria have different effects (11–16). Studies have shown that symbiotic bacteria can improve aphids’ heat tolerance and help them acclimate to high temperature environments (17, 18). It also has a significant impact on the aphids’ body color and resistance to diseases and parasitic wasps (19–21). In order for insects to adjust to the new host of nutrient utilization and metabolism, its symbiotic bacteria in the body can not only synthesize host the necessary amino acids, cholesterol, protein, and other nutrients, but also provide host decomposition of host plant cell wall enzymes, can also use the host plant secondary substances for detoxification, and can participate in the nitrogen cycle of the host, turning a host of nitrogen waste into nutrients (22, 23). It has been shown that *Aphis craccivora*, harboring *Arsenophonus* bacteria could better utilize *Robinia pseudoacacia* as a plant host. After inoculating *Arsenophonus* into an *A. craccivora* strain that lacked this bacterial endosymbiont and was incapable of colonizing *Robinia pseudoacacia*, the aphid strain acquired the ability to colonize *Robinia pseudoacacia* (24).

The research of symbiotic bacteria in cotton-specialized aphids, particularly in host transfer process, has not been documented, despite the fact that symbiotic bacteria of aphids are widely recognized. In this study, cotton-specialized aphids were transferred to and reared on an intermediate zucchini host for 10 generations, and changes in the diversity and abundance of aphid bacterial endosymbionts were assessed by sequencing the V3∼V4 region of the 16S rRNA gene using the Illumina MiSeq platform. We aimed to better understand how cotton-specialized aphids can adapt to cucumber hosts after being reared on an intermediate zucchini host at the level of aphid bacterial endosymbionts. This study contributes to the understanding of the relationship between the endosymbiotic bacterial composition of cotton-specialized aphids and their host specialization and shifting abilities.

## Materials and methods

### Plants and insects

Cotton (Zhong-mian49) and zucchini (Chunbaiyu) plants were used as the host plants in this study. They were grown in a growth chamber maintained at 26 ± 1°C, 75 ± 5% RH, and under a 14:10 h light/dark cycle. Cotton can be used to feed aphids when it reaches the 4-leaf stage.

Cotton-specialized aphids were collected from the experimental field station of the Institute of Cotton Research, Chinese Academy of Agricultural Sciences (CAAS) (36°5’34.8″N, 114°31’47.19″E) in May 2019. Cotton-specialized aphids were reared in petri dishes (diameter 9 cm) containing 1.8% agar and fresh host plant leaves, kept at 26 ± 1°C, 75 ± 5% relative humidity (20), under a 14:10-h light/dark photoperiod. *A. gossypii* biotypes were identified based on mitochondrial gene sequence polymorphisms described in the literature (25, 26). The identified cotton biotype aphids were maintained on cotton plants in the laboratory for over 40 generations (10 months) without exposure to any pesticides. Cotton-specialized aphids were transferred to and reared on zucchini leaves for 10 generations, and 3 day old aphids from each generation were collected. Four biological replicates were used for each generation.

### Sampling and DNA extraction

The control group (CK) was 3 day old cotton-specialized aphids that had only fed on cotton hosts; treatment groups consisted of 3 day old cotton-specialized aphids reared on the zucchini host from generations 1 to 10 (T1-T10). After collection, the 3 day old aphid samples were flash frozen in liquid nitrogen and stored at -80°C. Bacterial DNA was extracted from the aphid samples using the MagPure Stool DNA KF Kit B (Magen, China) according to the manufacturer’s instructions. DNA purity and concentration were determined via a Microplate Reader and Nanodrop 2000 spectrophotometer (Thermo Fisher Scientific, Carlsbad, CA, USA), respectively. The quality of the DNA samples was checked by 1% agarose gel electrophoresis.

### PCR amplification and library preparation

According to the specificity of the V3∼V4 variable region of the bacterial 16S rRNA gene, the degenerate PCR primers 515F (5’-GTGCCAGCMGCCGCGGTAA-3′) and 806R (5′-GGACTACHVGGGTWTCTAAT-3′) were used to amplify the DNA. Both forward and reverse primers were labeled with Illumina adaptor, pad, and adaptor sequences. PCR amplification was performed in a 50 μL reaction mixture containing 30 ng of DNA template. The PCR cycling conditions were 95°C for 3 min, 30 cycles of 95°C for 45 s, 56°C for 45 s, 72°C for 45 s, and a final extension for 10 min at 72°C. PCR products were purified using Agencourt AMPure XP beads and eluted in elution buffer. PCR libraries were quality checked using the Agilent Technologies 2100 Bioanalyzer (Agilent, USA), and qualified libraries were used for sequencing on the Illumina HiSeq 2500 PE250 platform (BGI, Shenzhen, China) according to protocols described by Gregory Caporaso and Eric J. de Muinck (27, 28), to generate 2 × 250 bp paired-end reads. Sequences obtained in this study have been deposited in GenBank^1^ under the accession number PRJNA764358.

### Sequencing and bioinformatics analysis

Raw reads were filtered to remove adapters, poly-N, and low quality (quality score < 20) sequences using the cutadapt v2.6 software. Filtered sequences were assembled into pairs by FLASH (Fast Length Adjustment of Short reads, v1.2.11) software (29), with the minimum overlap length set to 15 bp and the mismatch ratio of overlap regions ≤ 0.1, to obtain tags for the hypervariable region. The tags were clustered into OTUs (Operational Taxonomic Unit) at 97% sequence similarity using scripts in the USEARCH (v7.0.1090) software (30). UCHIME (v4.2.400) (31) was used to perform a reference-based chimera removal according to the gold database (v20110519) to obtain OTU abundance statistics tables for each sample. Finally, OTUs in each sample were classified using Ribosomal Database Project (RDP) Classifier 1.9.1 (32), using a minimum confidence threshold of 0.6 OTUs without annotation results; species that were not included in the analysis project were removed from the annotation results and the remaining OTUs were used for subsequent analysis. The community composition of each sample was calculated at the levels of phylum, class, order, family, genus, and species. Initially, the species abundance in all samples was less than 0.50% and all unclassified species were merged into “Others”. Alpha and beta diversity were estimated by MOTHUR (v1.31.2) (33) and QIIME (v1.80) (34) at the OTU level, respectively. PCoA (Principal coordinate analysis) was used to display differences between the samples based on the beta diversity distance matrix. Linear discriminant analysis (LDA) effect size (LEfSe) was used^2^ to analyze differences in relative abundance of OTUs between the different treatment groups and the control group. The LDA threshold value was set at >4 for treatment groups T1-T9, while that for treatment group T10 was set at 3.5. LDA score distribution histograms were developed using PERMANOVA to compare the differences in community structure between groups. For tests comparing only two groups, the Wilcoxon rank-sum test was used to show the significance of microbial community composition between different groups. If the number of groups was more than two, the Kruskal-Wallis Hest (kruskal. test in R) was used for multi-sample comparison. *P*-value <0.05 was used to assess the significance of differences.

## Results

### General description of 16S rRNA gene sequencing results

From sequences of 16S rRNA gene amplicon libraries from all ten generations (T1–T10) and control group (CK) had a total original reading of 1,641.93 Mbp before quality control (individual raw read numbers ranged from 60,461 to 299,627). A total of 3,106,199 paired reads were generated from 44 samples, with an average of 70,720 reads obtained for each sample (Table 1). After filtering, a total of 3,110,270 high-quality clean tags having an average length of 253 bp were obtained, with an average of 70,595 tags per sample. The percentage of bases with an effective sequencing error rate of <1% ranged from 99.94 to 99.97%. Reads for *A. gossypii* bacterial communities were produced, and 60 to 100 OTUs were identified (Table 1), with 97% identity cut-offs for each treatment group. A total of 10 phyla, 63 families, and 127 genera were annotated. Based on the observed species index, the sparse curves for all samples were almost saturated and stable, indicating that the 16S rRNA gene sequence was abundant and our analysis had sufficient depth to capture most of the microbial diversity information (Supplementary Figure 1).

**TABLE 1:** Sequencing analysis of 16S rRNA gene amplicons of *A. gossypii* with diversity indices.

Sample	No. of reads	OTUS*	Ace	Chao1	Shannon	Simpson	Good’s coverage (%)
**Diversity index**							
CK	89,982	100	116.73	113.70	1.35	0.46	99.97
T1	96,628	79	129.34	120.81	0.30	0.89	99.95
T2	65,970	73	118.76	108.19	0.37	0.86	99.96
T3	155,205	81	139.16	108.72	0.39	0.87	99.96
T4	115,867	75	123.60	107.96	0.44	0.85	99.96
T5	102,288	71	96.97	86.03	0.58	0.79	99.97
T6	131,514	67	104.24	90.63	0.92	0.64	99.96
T7	101,519	78	114.51	101.71	0.41	0.85	99.96
T8	111,234	60	92.58	82.52	0.36	0.87	99.97
T9	137,928	84	123.42	107.57	0.60	0.79	99.96
T10	115,817	86	116.52	102.94	0.49	0.83	99.96

*Operational taxonomic units (OTUs) were defined with pairwise 97% sequence identity.

### Effects of host transfer on the bacterial microbiota of *Aphis gossypii*

**FIGURE 1:**
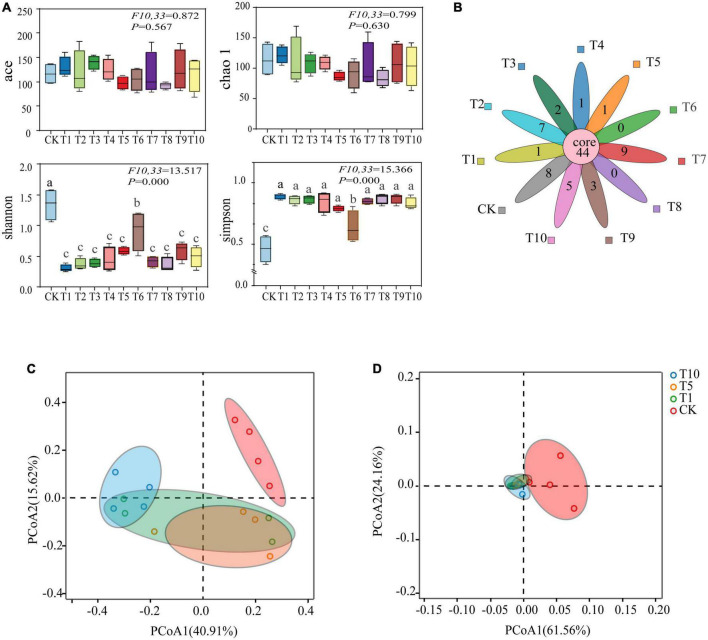
Sequencing analysis of 16S rDNA gene amplicons of cotton-specialized aphids with diversity indices. **(A)** Boxplot of α-diversity measured by the four indexs. **(B)** Core-Pan OTU presentation of the common and unique OTUs of all samples in the petal diagram. **(C)** Unweighted UniFrac metrics were used to determine pairwise distances among all of the samples. **(D)** Wighted UniFrac metrics were used to determine pairwise distances among all of the samples.

Good’s coverage, which estimates the percentage of the total species represented in a sample, averaged 99.96% thereby suggesting that the majority of microbial species present in *A. gossypii* were included in this study (Table 1). Alpha diversity analysis was used to determine the complexity of species diversity of each sample through several indices, including observed species, the diversity index of Ace, and Chao 1, all of which showed that there were significant differences in Shannon and Simpson indexes among the control and treatment groups (*P* < 0.05), demonstrating that microbial diversity and evenness decreased among treatments (Figure 1A). Petal plots showed that there were 44 common OTUs in the control (CK) and all treatment (T1-T10) groups, and T7 had the most OTUs. In contrast to the 8 unique OTUs in the control (CK) group, T6 and T8 had the least number and no unique OTUs (Figure 1B). In the PCoA analyses based on species level OTU, points that are close together represent samples that were highly similar in their community composition. Representative T1, T5, and T10 were selected from the treatment group (T1-T10) for comparison with CK and the results showed that the bacterial communities of CK and the selected treatment groups were separated (Figures 1C, D), and that difference between were statistically significant (*P* < 0.001).

### Phylum level comparisons of bacterial communities from different generations of aphids reared on different plant hosts

**FIGURE 2:**
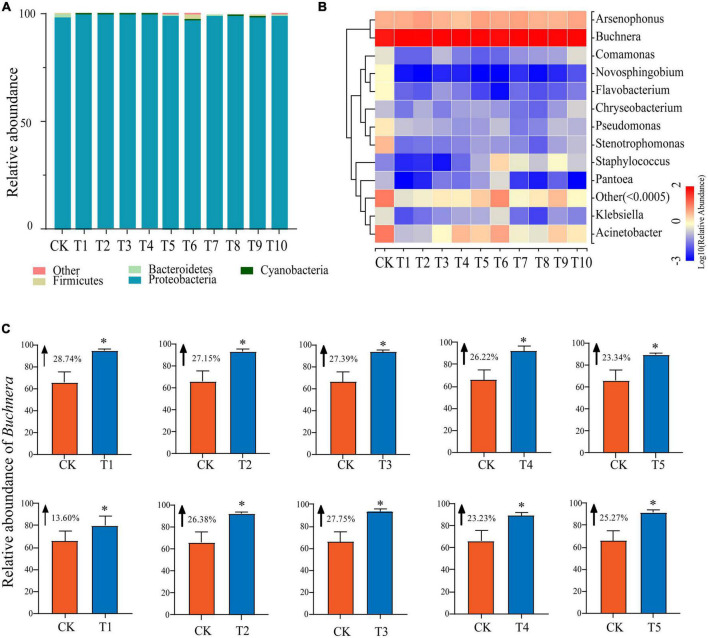
Changes in relative abundance of symbiotic bacteria in cotton-specialized aphids fed on zucchini for 1 to 10 generations and the control group (CK). **(A)** Relative abundance of symbiotic bacteria in cotton-specialized aphids fed on zucchini for 1 to 10 generations and the control group (CK) at the phylum level. **(B)** Relative abundance of symbiotic bacteria in cotton-specialized aphids fed on zucchini for 1 to 10 generations and the control group (CK) at the genus level. **(C)** Transfer to the Zucchini host significantly affected the relative abundance of *Buchnera* in the microbial communities of cotton-specialized aphids. Pairwise comparison between the treatment group and CK, the data is mean ± SD. *P < 0.05.

To better understand differences between the microbial communities in *A. gossypii* aphids before and after host transfer, we classified the microbial communities of the control (CK) and treatment (T1-T10) groups at the phylum level (Supplementary Table 1 and Figure 2A). Among them, the dominant phyla were *Proteobacteria*, *Firmicutes*, and *Bacteroidetes*, with the abundance of *Proteobacteria* being highest regardless of host transfer (average 99.03% of all samples). The relative abundance of *Proteobacteria* was lowest in the T6 group (97.31%) and highest in the T1 group (99.70%). There were no significant differences in relative abundance between the treatment and control groups, or between the individual treatment groups (T1-T10). As for *Firmicutes*, the relative abundance fluctuated steadily in groups T1-T4 and was mostly consistent with CK (0.05%), however the abundance began to shift in group T5 (0.14%) and reached its maximum in group T6 (1.97%) which was significantly different (*P* < 0.05) than the other groups. The relative abundance of *Firmicutes* in groups T7-T10 was slightly higher than CK. Compared with CK (1.39%), the mean relative abundance of *Bacteroidetes* was significantly (*P* < 0.05) lower in groups T1-T10 but there was no significant difference between the individual treatment groups.

### Genus level comparisons of bacterial communities from different generations of aphids reared on different plant hosts

At the genus level, the most abundant genera (relative abundance > 1.00%) in the CK group were *Buchnera* (66.08%), *Acinetobacter* (11.28%), *Arsenophonus* (3.95%), *Stenotrophomonas* (3.41%), *Pseudomonas* (1.42%), *Flavobactera* (1.03%), and *Novosphingobium* (1.02%) (Supplementary Table 2 and Figure 2B). Compared with CK, the top three most abundant genera in the treatment groups were *Buchnera*, *Acinetobacter*, and *Arsenophonus*. Excluding *Buchnera* and *Arsenophonus*, the abundance of other symbiotic bacteria decreased significantly (*P* < 0.05) after plant host transfer, and the abundance of *Flavobacterium* and *Novosphingobium* approached 0. There were no significant differences between individual treatment groups. The relative richness of *Arsenophonus* fluctuated significantly among T1-T10 treatment groups, among which T5 (5.33%) was the largest and T4 (2.78%) was significantly smaller than CK (3.95%) (*P* < 0.05). *Buchnera*, as the dominant genera in CK, persisted in the treatment groups (T1-T10). In addition, all treatment groups (T1-T10) were significantly higher than the control group CK (66.08%) (*P* < 0.05), and reached the highest value at T1 (94.82%) (Figure 2C).

### Comparisons of biomarkers from microbial communities of different generations of aphids reared on different plant hosts

**FIGURE 3:**
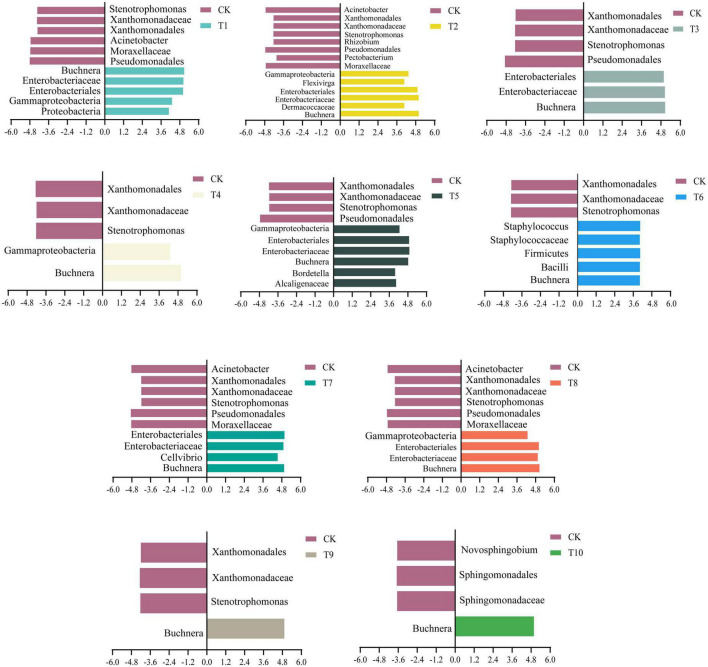
Comparison of biomarkers of different generations of cotton-specialized aphids on cotton host (CK) and transferred to zucchini host (T1-T10).

LEfSe analysis was performed to screen for biomarkers in aphid bacterial communities (Figure 3). A different number of biomarkers were identified in the treatment group compared with the control (LDA > 4, only T10 LDA > 3.5). It was found that as the number of progenies of the aphid population increased, the specific microbial biomarkers decreased in each generation at specific LDA thresholds until only *Buchnera* was found in the T9 and T10 generations of aphids. And *Buchnera* was found in the whole generation T1-T10 of zucchini hosts. These results suggest that *Buchnera* may play an important role in the adaptation of cotton aphids to the intermediate zucchini host.

## Discussion

**FIGURE 4:**
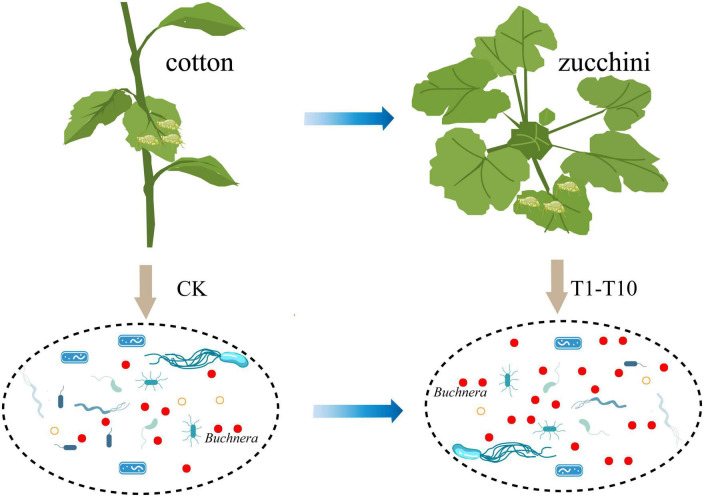
Pattern of transfer of cotton-specialized aphids from cotton to zucchini.

Biotypes represent excesses in the evolutionary process of species. In insects, most biotypes are classified according to the ability to use different hosts (34). However, the presence of an intermediate host, zucchini, suggests that specialization is not absolute and that some endosymbiotic bacteria provide essential substances to compensate for the nutritional maladjustment of the host’s diet (8). Therefore, this study reveals for the first time the transformation mechanism of cotton specialized aphids using zucchini as an intermediate host from the perspective of symbiotic bacteria (Figure 4). The results showed that the presence of *Buchner*’s provided an important role for the transformation of aphids in plant hosts.

It is well known that almost all insects contain symbiotic bacteria, which form mutually beneficial relationships with their aphid hosts in different ways. Hemiptera cannot produce cholesterol, vital amino acids, or vitamins, and the nutrients they take in from plant sap are insufficient and imbalanced, according to studies on the roles of symbiotic bacteria (23). While Primary symbiotic bacteria would play their role to provide nutrients. However a large number of studies have shown that Secondary symbiotic bacteria can also affect the growth and development, reproductive ability, and environmental adaptation of their host insects (35, 36). According to the research by Sasaki et al., the symbiotic yeast bacteria in *Nilaparvatalugens*, the primary pest of rice, have high activity of uricase, which may use the metabolic waste (uric acid) of the host to produce amino acids needed for *Nilaparvatalugens*’ growth and development (37).

In our study, the microbiota of *A. gossypii* was dominated by a few bacterial taxa both before and after host transition. At the phylum level, *Proteobacteria* and *Firmicutes* were the dominant taxa, with the abundance of *Proteobacteria* being highest at 99.09%, which is similar to that found in other hemipteran insects (38, 39) (Figure 2A). *Firmicutes* and *Proteobacteria* are essential to maintaining the growth and development of insects, aiding in the metabolism of secondary metabolites from host plants. *Bacteroidetes* are often the dominant bacterial phylum in the intestinal microbe communities of many insects such as the pea aphid in order Hemiptera and the longhorn beetle in Coleoptera, which can degrade plant cell wall polysaccharides (40, 41). The third major community in this study’s cotton aphids was the *Bacteroidetes*, and when zucchini was used as the host plant, the population’s richness drastically fell. Therefore, it may be assumed that the decline in *Bacteroidetes* may make it difficult for aphids to efficiently enter the cell wall for effective nutrition while feeding on zucchini. *Actinobacteria* can promote the growth and development, and improve the resilience of their insect hosts (42, 43). The lack of actinomycetes in aphids that we discovered in this research, leads us to believe that the aphids’ immunity may have been diminished as a result of the transition in plant hosts, and also that the change in host may have increased aphid maladaptation. In addition, the species diversity of the microbial community in cotton-specialized aphids decreased significantly after plant host transfer. Such results are consistent with the results of Mottal et al. who showed a low diversity of symbiotic bacteria in environmentally sensitive honey bees, i.e., the lower the diversity of symbiotic bacteria, the weaker the resistance (44, 45).

At the genus level, a variety of microbial communities were detected in the aphids, and there were significant differences in microbial communities between aphids feeding on cotton and zucchini hosts. The dominant bacteria in the microbial communities of cotton-specialized aphids fed on zucchini mainly consisted of primary symbiotic bacteria *Buchnera* and secondary symbiotic bacteria *Arsenophonus* and *Acinetobacter* (Supplementary Table 2 and Figure 2B). Studies have shown that only 11 of the 20 amino acids required for aphids to develop can be produced in the body, with the other nine coming from plants. The fitness of cotton aphids will be diminished if one of the required amino acids is lacking or the host plant’s supply is very low, which prevents the plant from providing what the aphids need to grow and develop (22). Douglas discovered that *Buchnera* in pea aphid may give a range of important amino acids to pea aphid, enabling the host to overcome the nitrogen nutritional barrier of plants (46, 47). By examining the whole genome sequence of the pea aphid, Consortium et al. discovered that *Buchnera* and the aphids are closely related in terms of nutrient metabolism because they share metabolic pathways for certain important amino acids (48, 49). Our study found that as the dominant species present throughout the host shifting process, the relative abundance of *Buchnera* significantly increased in cotton-specialized aphids that had been reared on zucchini for 10 generations compared with those reared on cotton (CK). This confirms the important role of *Buchnera* in host transfer, i.e., when aphids encounter host plants that are not very favorable to them, they increase the symbiotic bacteria. We speculate that it exercises a specific function to regulate the metabolism of nutrients to improve adaptation. It also confirms that endosymbiotic bacteria of aphids mediated by intermediate plant hosts can alter aphid specialization, and the specialization is not a dead-end in the evolution of feeding habit.

Studies have also found that the secondary symbiotic bacteria *Arsenophonus* plays an important role in resisting natural enemies, expanding the host range, and regulating the reproduction of its aphid host. Infection with of the soybean aphid with *Arsenophonus* bacteria can help improve the aphid’s ability to adapt to different plant hosts, and promoted growth of the aphid population (50). *Acinetobacter* was involved in many life activities of aphid host, including food digestion, nitrogen transformation and assimilation, and degradation of harmful substances (51, 52). Other endosymbiotic bacteria, including *Stenotrophom*, *Pseudomons*, *Flavobacterium*, and *Novosphingobium*, were likewise considerably reduced in the cotton aphid feeding on zucchini, and even their relative richness was close to 0. These further demonstrated the reduction in species variety caused by the transfer of cotton-specific cotton aphid to the host of zucchini. The same study also pointed out that there are complex relationships based on resources, survival niches, and interactions between aphids and bacterial symbionts, known as the metabolic tug of war (53). *Arsenophonus* infection may have an impact on *Buchnera* richness, which rose in correlation with *Arsenophonus* richness (54), whereas *Hamiltonian* exhibited the reverse connection (25). None of these interactions, nevertheless, were discovered in our analysis.

In summary, endosymbiotic bacteria, particularly *Buchnera*, played a significant role in nutrition absorption during the transplantation of cotton-specialized aphid to intermediate host zucchini, while other secondary symbiotic bacteria influenced probably more the immunological aspects of cotton-specialized aphid. In other words, the cotton-specialized aphid was not fully adaptation to the intermediate host, zucchini, and it could only improve its adaptation by expanding its own *Buchnera*. This behavior accelerates the spread of aphids and increases their damage, while avoiding the problem of direct transfer of cotton aphids to poorly adapted hosts such as cucumber due to nutritional deficiencies. However, in general, identifying the significance of intermediate hosts and symbiotic bacteria is only the first step in controlling cotton aphids and in understanding the transformation mechanisms of cotton aphids, and more research is needed to understand the transformation mechanisms of symbiotic bacteria.

## Conclusion

To our knowledge, this is the first study of the dynamics of endosymbiotic bacteria in specialized aphids when zucchini act as intermediate hosts. We examined the changes of endosymbiotic bacteria before and after the transfer of aphids from cotton to zucchini host. It was found that the intermediate zucchini host affected bacterial species richness of cotton aphid microbial communities, but did not affect the structural composition of bacterial species. In addition, the dominant bacterial species in *A. gossypii* were *Buchnera* and *Arsenophonus*. In particular, the changes in *Buchnera* abundance were the most obvious. *Buchnera* was not only the dominant population before host transformation, but also significantly increased after *A. gossypii* were transferred to zucchini. This phenomenon confirms the importance of *Buchnera* in the host transfer process of cotton-specialized aphids. Therefore, we speculate that *Buchnera* may contribute to aphid-altered specialization through zucchini in a nutrient-providing manner. Our study reveals a complex relationship between symbiotic bacteria in the host transfer ability of *A. gossypii* and provides a theoretical basis for the mechanism of *A. gossypii* specialization type alteration.

## Data availability statement

The datasets presented in this study can be found in online repositories. The names of the repository/repositories and accession number(s) can be found below: https://www.ncbi.nlm.nih.gov/, PRJNA764358 (SRA).

## Author contributions

XG, JC, and JLu conceived and designed the experiments. WX and WL analyzed the data. DL, XZ, LW, JJ, and XG evaluated the conclusions. WX, JLi, and WL wrote the manuscript. All authors read and approved the final manuscript.

## Footnotes

1
http://www.ncbi.nlm.nih.gov

2
http://huttenhower.sph.harvard.edu/lefse

## Supplementary material

Supplementary Figure 1 Rarefaction curves based on species abundance data.
