# Supplementary material for: Buchnera breaks the specialization of the cotton-specialized aphid (Aphis gossypii) by providing nutrition through zucchini
Source: Front Nutr. 2023 Mar 21;10:1128272. doi: 10.3389/fnut.2023.1128272 (PMC10071829; doi:10.3389/fnut.2023.1128272)
Supplement: Supplementary file 1 [file Data_Sheet_1.docx]

**Supplementary Table**

**Table S1. Relative abundance of symbiotic bacteria in cotton-specialized aphids reared on Zucchini for 1 to 10 generations and the control group (CK) at the phylum level.**

**Table S2. Relative abundance of symbiotic bacteria in cotton-specialized aphids reared on Zucchini for 1 to 10 generations and the control group (CK) at the genus level.**

**Table S1. Relative abundance of symbiotic bacteria in cotton-specialized aphids reared on Zucchini for 1 to 10 generations and the control group (CK) at the phylum level.**

| Groups | *Proteobacteria* (%) | *Bacteroidetes* (%) | *Firmicutes* (%) |
| --- | --- | --- | --- |
| CK | 98.40±1.40 a | 1.39±1.42 a | 0.05±0.04 b |
| T1 | 99.70±0.12 a | 0.11±0.06 b | 0.02±0.01 b |
| T2 | 99.53±0.29 a | 0.27±0.24 b | 0.03±0.03 b |
| T3 | 99.51±0.19 a | 0.18±0.09 b | 0.01±0.02 b |
| T4 | 99.57±0.13 a | 0.20±0.11 b | 0.03±0.03 b |
| T5 | 99.03±0.41 a | 0.19±0.08 b | 0.14±0.18 b |
| T6 | 97.31±2.00 a | 0.14±0.08 b | 1.97±1.68 a |
| T7 | 99.06±0.71 a | 0.12±0.17 b | 0.64±0.76 b |
| T8 | 99.43±0.14 a | 0.05±0.09 b | 0.29±0.11 b |
| T9 | 98.70±0.85 a | 0.19±0.16 b | 0.91±0.86 b |
| T10 | 99.06±0.86 a | 0.46±0.78 b | 0.31±0.79 b |

* Statistical significance based on one-way ANOVA tests, data in the present table are mean ± SD. Values in the same column followed by different letters are significantly different at *P*<0.05.

**Table S2. Relative abundance of symbiotic bacteria in cotton-specialized aphids reared on Zucchini for 1 to 10 generations and the control group (CK) at the genus level**

| Groups | *Buchnera* | *Arsenophonus* | *Acinetobacter* | *Stenotrophomonas* | *Pseudomons* | *Flavobacterium* | | *Novosphingobium* |
| --- | --- | --- | --- | --- | --- | --- | --- | --- |
| CK(%) | 66.08±9.33 c | 3.95±0.86 ab | 11.28±11.90 a | 3.41±5.74 a | 1.42±1.95 a | 1.03±1.22 a | 1.02±1.91 a | |
| T1(%) | 94.82±1.29 a | 4.22±0.91 ab | 0.17±0.20 b | 0.02±0.01 b | 0.21±0.25 b | 0.02±0.01 b | 0.00±0.00 b | |
| T2(%) | 93.23±2.13a | 5.53±1.55 a | 0.21±0.10 b | 0.03±0.00 b | 0.15±0.11 b | 0.01±0.01 b | 0.00±0.00 b | |
| T3(%) | 93.47±1.92a | 3.64±1.08 ab | 1.05±1.35 b | 0.03±0.01 b | 0.11±0.10 b | 0.07±0.08 b | 0.00±0.00 b | |
| T4(%) | 92.30±4.56 a | 2.78±0.58 c | 3.25±3.76 b | 0.04±0.01 b | 0.06±0.03 b | 0.03±0.03 b | 0.00±0.00 b | |
| T5(%) | 89.42±1.25 a | 5.33±0.94 a | 2.24±1.07b | 0.08±0.08 b | 0.07±0.05 b | 0.01±0.00 b | 0.00±0.00 b | |
| T6(%) | 79.69±8.63 b | 4.58±1.92 ab | 4.97±4.97 b | 0.06±0.02 b | 0.20±0.36 b | 0.00±0.00 b | 0.00±0.00 b | |
| T7(%) | 92.46±1.41 a | 5.19±0.61a | 0.70±0.64b | 0.04±0.05 b | 0.03±0.01 b | 0.02±0.02 b | 0.00±0.00 b | |
| T8(%) | 93.83±2.34 a | 3.85±0.62 ab | 0.50±0.73b | 0.01±0.02 b | 0.04±0.06 b | 0.01±0.02 b | 0.00±0.00 b | |
| T9(%) | 89.31±2.83 a | 3.65±0.67 ab | 2.41±1.34 b | 0.09±0.06 b | 0.18±0.20 b | 0.01±0.02 b | 0.00±0.00 b | |
| T10(%) | 91.35±2.72 a | 4.78±1.68 a | 1.44±1.36 b | 0.18±0.35 b | 0.14±0.03 b | 0.03±0.03 b | 0.01±0.02 b | |

* Statistical significance based on one-way ANOVA tests, data in the present table are mean ± SD. Values in the same column followed by different letters are significantly different at *P*<0.05.
