# Supplementary figures and images for: Buchnera breaks the specialization of the cotton-specialized aphid (Aphis gossypii) by providing nutrition through zucchini
Source: Front Nutr. 2023 Mar 21;10:1128272. doi: 10.3389/fnut.2023.1128272 (PMC10071829; doi:10.3389/fnut.2023.1128272)

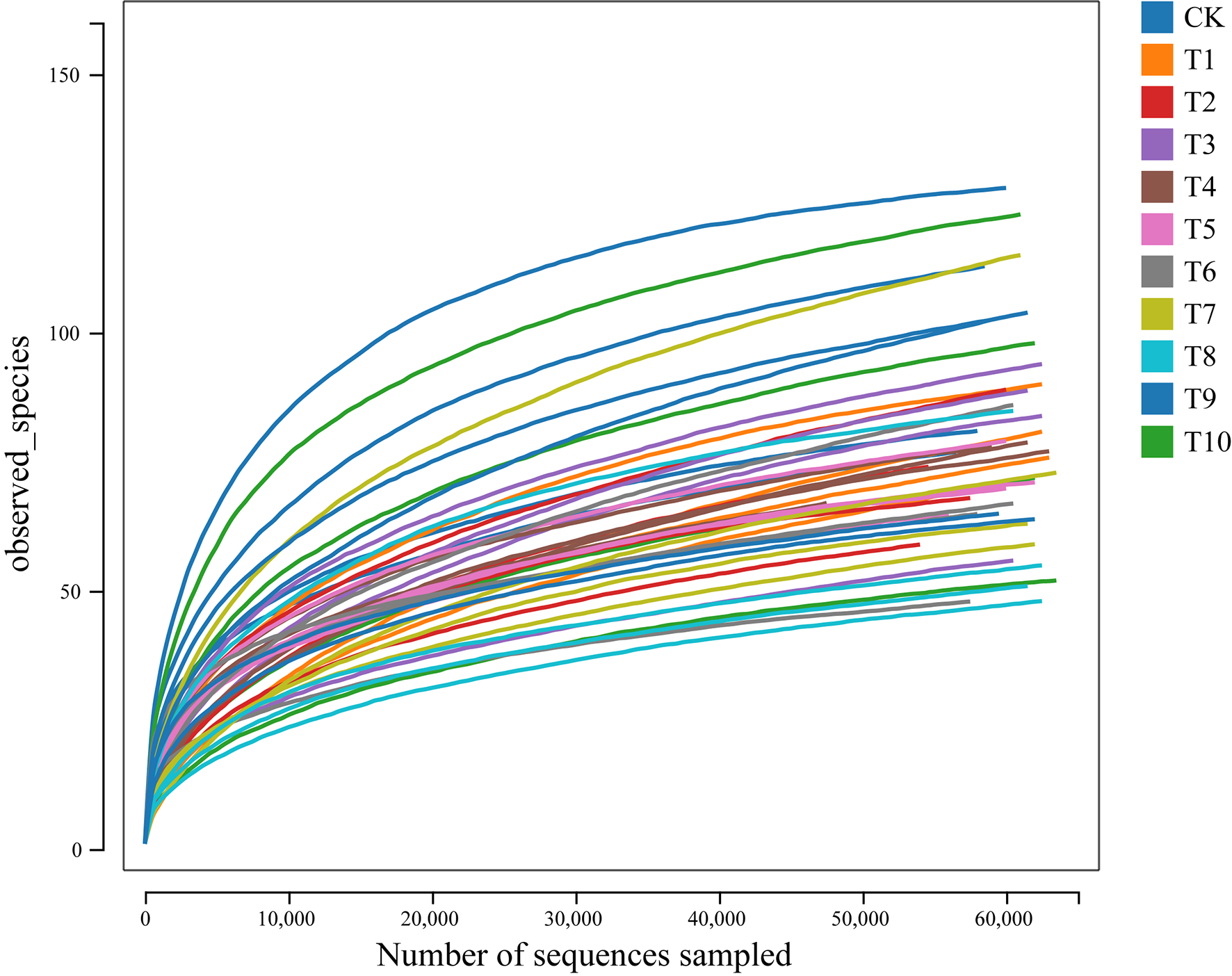

Supplement: Supplementary Figure 1 — Rarefaction curves based on species abundance data. [file Image_1.TIF]
